# Telomere length and body temperature—independent determinants of mammalian longevity?

**DOI:** 10.3389/fgene.2013.00111

**Published:** 2013-06-13

**Authors:** Gilad Lehmann, Khachik K. Muradian, Vadim E. Fraifeld

**Affiliations:** ^1^The Shraga Segal Department of Microbiology, Immunology, and Genetics, Center for Multidisciplinary Research on Aging, Ben-Gurion University of the NegevBeer Sheva, Israel; ^2^Institute of Gerontology of the National Academy of Medical Sciences of UkraineKiev, Ukraine

## Why do species differ in lifespan and what are the determinants of their longevity?

Understanding the main factors that determine variation in species longevity may provide a clue into the leading mechanisms of aging and—what is even more important—outline the key targets for longevity-promoting interventions. Comparative studies on longevity in mammals revealed numerous variables that correlate with maximum lifespan (MLS). However, because of the intangibly intertwined biological relationships, only a limited number of the variables could be considered independent determinants of longevity. Most other correlations reflect intermediated (formal) co-variations rather than the “cause-and-effect” links. It is obvious that manipulations of the formal correlates have little chances to effect the longevity-ensuring systems, and thus be helpful for developing experimental strategies of lifespan extension. Therefore, in comparative longevity studies, it is important to discriminate the independent determinants from the formal correlates.

One of the simple criteria to distinguish determinants of longevity is consistently high correlation of a given variable with MLS observed in various model systems. The other approach is based on more ambiguous statistical methods, multivariate analyses included (see, for example, Lehmann et al., 2008a). In particular, we have previously shown that body mass (BM) or resting metabolic rate alone explain around 40–50% of the variation in mammalian longevity, whereas their combination with mitochondrial DNA (mtDNA) GC content could explain over 70% of the MLS variation (Lehmann et al., 2008a,b). Consequently, we hypothesized that other putative players in MLS determination should have relatively small contribution or their effects should be mediated by the above factors.

Recent finding by Gomes et al. (2011) demonstrating a strong negative correlation (*p* = 0.0032) between telomere length and MLS in 59 mammalian species calls for re-evaluation of this hypothesis. Indeed, the coefficient of MLS determination (*R*^2^) calculated using the data in their paper indicates that the telomere length could alone explain more than 1/3 of the variation in the lifespan of mammals. Here, we explore whether the telomere length has an independent impact on mammalian longevity or its effect is attributed to co-variation with other determinants of MLS, such as BM and mtDNA GC content. Our analysis was based on the set of mammalian species with the telomere length records presented in Gomes et al. (2011) (*n* = 55; four species from the orders Monotremata and Diprotodontia, with unusually short telomeres of 1 kb, were not included in the analysis). The MLS, BM, and body temperature (*T*_*b*_) records were retrieved from the AnAge database (Tacutu et al., 2013; http://genomics.senescence.info/species/). Calculation of the mtDNA GC content was described elsewhere (Lehmann et al., 2008a). To ensure linear relationships, MLS and BM values were ln-transformed, i.e., MLS was presented as a natural logarithm of MLS (lnMLS) and BM was presented as a natural logarithm of BM (lnBM).

Re-evaluation of combined effect of lnBM and mtDNA GC on lnMLS in the set of species analyzed by Gomes et al. (2011) gave an extremely significant coefficient of MLS determination (*R*^2^ = 0.713, *P* = 6.2E-10, *n* = 37), which is very close to that obtained on much bigger dataset of mammals (*R*^2^ = 0.703, *P* < E − 25, *n* = 215; unpublished data). The telomere length significantly correlates with both lnBM (*R* = −0.479, *P* = 0.00025, *n* = 55) and mtDNA GC content (*R* = −0.44, *P* = 0.006, *n* = 37), suggesting that the relationship between lnMLS and telomere length (*R* = −0.609, *P* = 1.2E-10, *n* = 54) could, at least in part, be due to the telomere length association with lnBM and/or mtDNA GC. Nevertheless, partial correlation and multivariate analyses showed that the telomere length has an independent impact on longevity determination.

The partial correlation analysis allows eliminating the co-variation effects. We found that the correlative links between telomere length and BM or mtDNA GC do not significantly alter its association with longevity. Indeed, removing the effects of lnBM or GC or both did not affect the correlation between lnMLS and telomere length (coefficients of partial correlations are −0.421, −0.608, and −0.442, respectively; *P* < 0.01). Moreover, the links between telomere length and lnBM or mtDNA GC are apparently mediated via longevity-associated factors because removing the effect of MLS resulted in an insignificant correlation of telomere length with lnBM (*P* > 0.5) or GC content (*P* > 0.4).

**Figure 1:**
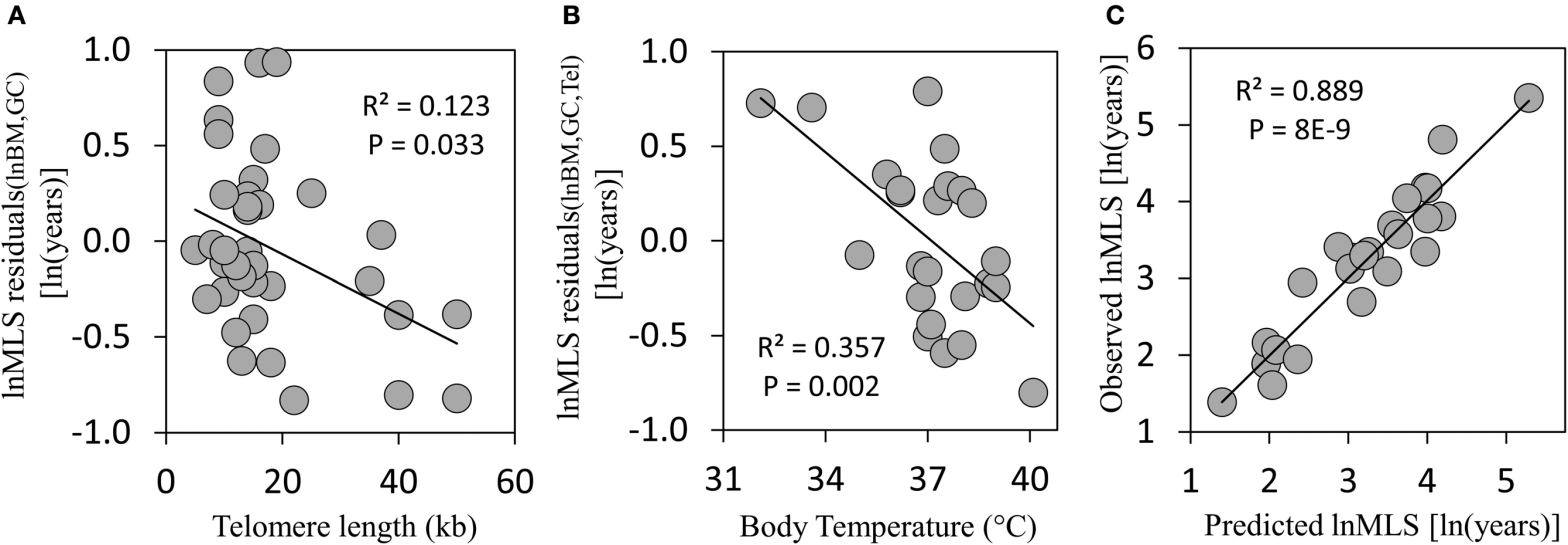
**(A)** The lnMLS residuals of lnBM and mtDNA GC correlate with telomere length (Tel). **(B)** The lnMLS residuals of lnBM, mtDNA GC, and Tel correlate with *T*_*b*_. **(C)** Observed lnMLS plotted against predicted lnMLS. The predicted lnMLS was calculated from the multivariate linear regression: lnMLS = 0.14lnBM + 0.015mtDNA GC − 0.017Tel − 0.17 *T*_*b*_, where MLS is in years, BM in g, mtDNA GC content in b/kb, Tel in bp, and *T*_*b*_ in °C. Statistical calculations were performed using the statistical package for the social sciences (SPSS, Inc., Chicago, IL) software.

As further demonstrated in Figure 1A, after extraction of lnBM and mtDNA GC input, the lifespan residuals significantly correlate with telomere length (*R* = 0.351, *P* = 0.033). The multivariate analysis showed that the telomere length together with lnBM and mtDNA GC determine 76.9% (*P* = 1.3E-10) of lnMLS variation, thus increasing the lnMLS *R*^2^-value (71.3%) of two variables, lnBM and mtDNA GC, by 5.6%. That is, the telomere length could explain part of the variation in the mammalian longevity, which is not explained by the lnBM and mtDNA GC. However, there is still a place for unaccounted factors as the lnMLS residuals of lnBM, mtDNA GC, and telomere length significantly correlated with lnMLS (*R* = 0.481, *P* = 0.003). In attempt to discover these still unaccounted factors, we further included in the analysis an additional variable closely related but not identical to the metabolic rate—body temperature (*T*_*b*_).

Gomes et al. (2011) hypothesized that the evolution from exothermic to homeothermic organisms was accompanied by telomere shortening as a tumor protective adaptation to an enhanced mutation load caused by high *T*_*b*_. Yet, within mammalian species we did not observe any significant correlation between the telomere length and typical *T*_*b*_ (*P* > 0.8, *n* = 37). There was also no significant correlation (*P* > 0.3) between *T*_*b*_ and lnMLS.

Unexpectedly, we found that *T*_*b*_ significantly correlates with the residual lifespan of lnBM, mtDNA GC and telomere length (*R* = 0.597, *P* = 0.002; Figure 1B). This may explain some cases of considerable deviations from the lnMLS predicted by lnBM, mtDNA GC, and telomere length. For example, the naked mole-rat (*Heterocephalus glaber*) and North American pika (*Ochotona princeps*) have similar values of lnBM, mtDNA GC content and telomere length, yet the naked mole-rat lives 4.4 times longer. This apparent “discrepancy” could be largely attributed to the difference in *T*_*b*_ which, in the sample analyzed, is the lowest for the naked mole-rat (32.1°C) and the highest for the North American pika (40.1°C). Of note, the lnMLS residuals of all four variables (lnBM, mtDNA GC, telomere length, and *T*_*b*_) did not correlate significantly with lnMLS (*P* = 0.112). As a result, Figure 1C demonstrates extremely high fitting between predicted and observed lnMLS values (*R*^2^ = 0.889, *R* = 0.943, *P* = 8.0E−9). Thus, in the analyzed set of mammalian species, the combination of lnBM, mtDNA GC, telomere length, and *T*_*b*_ explains the vast majority of lnMLS variation. The remaining variation of about 11.1% is most likely attributed to the “noise” in measuring of the above variables. Yet, an input of still unaccounted factors or cross-talk effects cannot be dismissed and continued study is warranted. For example, the interactions within the telomeres—p53—mitochondria axis (Sahin and DePinho, 2012), could be an important area for future search.

The results obtained gained further support from the multivariate analysis with standardized coefficients showing a significant impact of each of the variables under analysis, i.e., lnBM (*P* = 2.6E-6), mtDNA GC (*P* = 0.0007), telomere length (Tel; *P* = 0.032) and *T*_*b*_ (*P* = 0.0015), on lnMLS determination:

$$\text{lnMLS}=0.586\text{lnBM}+0.356\text{mtDNA GC}-0.230\text{Tel}-0.294T_{b}$$

**Figure 2:**
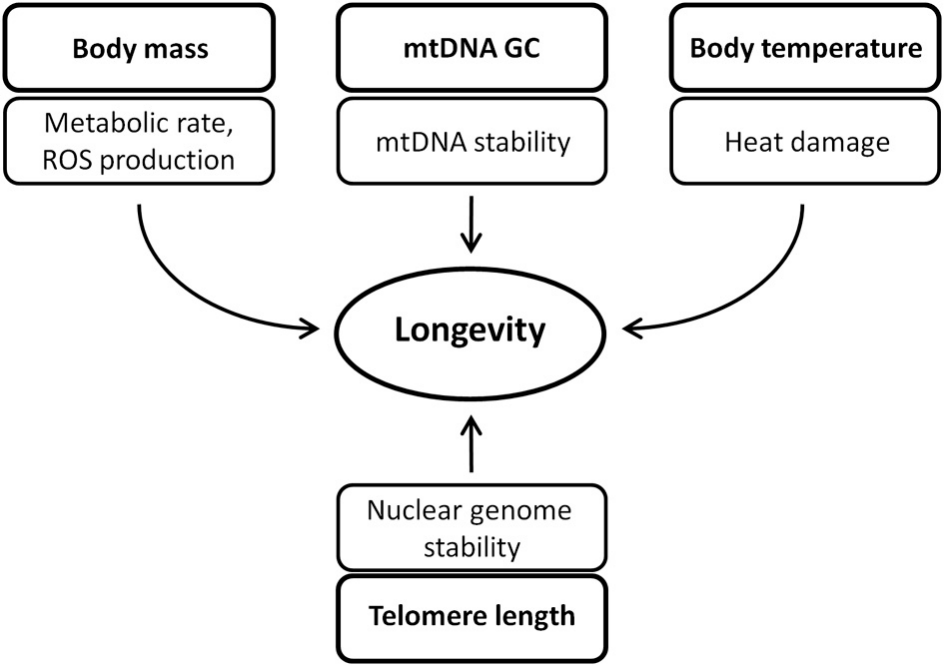
**Determinants of mammalian longevity.** In the examined set of species, the relative contribution to MLS determination decreases in a descending order: Body mass > mtDNA GC content > Body temperature > Telomere length. Of note, 3 of 4 determinants (upper panel) are directly associated with mitochondria. For explanations, see the text.

As could be expected, lnBM and mtDNA GC content display positive coefficients of regression while telomere length and *T*_*b*_ display negative coefficients, highlighting the role of potentially damaging and protective (stabilizing) factors in lifespan determination (Figure 2). The higher BM is associated with a lower metabolic rate and lesser generation of damaging substances (e.g., ROS). The higher GC content may ensure the higher thermodynamic stability of mtDNA against denaturizing factors such as high *T*_*b*_. While short telomeres have less probability to be damaged than longer ones, their maintenance and efficient repair are of crucial importance for chromosome and genome stability. It seems plausible that the age-related reduction in telomere length is more pronounced in short-lived than in long-lived species. This notion gained experimental support from a recent observation of Vera et al. (2012), who showed that the mouse telomeres shortened 100 times faster than human telomeres. The shorter (but more stable) telomeres in long-lived/large mammals evolved apparently due to more efficient DNA repair [reviewed by Moskalev et al. (2012)], along with a prominent reduction in telomerase activity with an increase in BM (Seluanov et al., 2007; Gomes et al., 2011). An important observation is that mammalian species (large rodents, humans) that use replicative senescence, a potential tumor suppression mechanism, have also relatively short telomeres (Seluanov et al., 2008). Altogether, the increase in BM and mtDNA GC content, reduction in telomere length and lower *T*_*b*_ could result in a higher genomic and metabolic stability, more efficient cellular homeostasis, and ultimately in increased longevity (Figure 2).
